# Administration of Young Coconut (*Cocos nucifera* L.) Juice Ameliorates Memory Impairment in a Menopausal Rat Model

**DOI:** 10.3390/diseases12100250

**Published:** 2024-10-12

**Authors:** Saeko Sugiyama, Hiroshi Matsushita, Akira Minami, Hatsune Nakao, Shota Hata, Ayumi Matsumoto, Hideyuki Takeuchi, Akihiko Wakatsuki

**Affiliations:** 1 Department of Obstetrics and Gynecology, School of Medicine, Aichi Medical University, Nagakute 480-1195, Aichi, Japan; 2 Department of Biochemistry, School of Pharmaceutical Sciences, University of Shizuoka, Suruga-ku, Shizuoka 422-8526, Shizuoka, Japan; a.minami.da@juntendo.ac.jp (A.M.); htakeuchi@u-shizuoka-ken.ac.jp (H.T.)

**Keywords:** coconut, depression, memory impairment, menopause, ovariectomy, rat

## Abstract

Background/Objectives: In Southeast Asia, the traditional use of young coconut (*Cocos nucifera* L.) juice (YCJ) by women to alleviate postmenopausal symptoms suggests potential estrogenic properties. However, few studies explore the impact of YCJ on pathologies associated with estrogen deficiency in postmenopausal animal models. This study examines the impact of YCJ supplementation on memory impairment and depression-like behavior in ovariectomized (Ovx) rats. Methods: Ten-week-old female rats underwent either a sham operation (Sham) or bilateral Ovx. The rats in the Ovx + YCJ group received 5×-concentrated YCJ by gavage at a dose of 15 mL/kg body weight. Twelve weeks later, the Morris water maze and forced swim tests were used to evaluate hippocampus-dependent spatial memory and depression-like behavior, respectively. Results: The Ovx rats displayed significant memory impairment (*p* < 0.05) and depression-like behaviors (*p* < 0.05), while the memory performance in the rats in the Ovx + YCJ group resembled that of the Sham rats. However, the administration of YCJ did not result in the improvement of depression-like behavior. Conclusions: These findings suggest that YCJ consumption may help ameliorate memory impairment in postmenopausal women.

## 1. Introduction

Most women experience menopause between the ages of 45 and 54; it is a stage marked by substantial hormonal shifts and the cessation of the menstrual cycle [1]. The depletion of sex steroid hormones associated with menopause potentially increases vulnerability to age-related conditions in various hormone-responsive tissues, such as brain, bone, and the cardiovascular system [2], resulting in women becoming susceptible to chronic diseases after menopause, including Alzheimer’s disease [3], osteoporosis [4], and cardiovascular disease [5].

Menopausal hormone therapy (MHT) was once widely embraced for its role in preventing or treating such age-related diseases. However, two decades ago, findings from the Women’s Health Initiative revealed that the risks associated with MHT outweighed its benefits [6,7]. To date, re-analysis of the results has revealed that MHT remains effective for the alleviation of vasomotor symptoms, preserving bone density and reducing fractures in women under 60 or those undergoing MHT within 10 years of menopause [8,9,10]. In addition, a recent systematic review highlighted favorable outcomes in all-cause mortality, cardiac mortality, and coronary heart disease events among women under 60 who initiated MHT [11]. However, candidates for MHT continue to hesitate before initiating MHT because of the associated risks. As a result, there is growing interest in complementary and alternative therapies to alleviate the symptoms associated with menopause [1]. Against this background, we ultimately aim to establish alternatives to MHT for preventing age-related diseases in postmenopausal women. Thus, we preliminarily tested the effects of several natural products on menopause-associated pathologies using a menopausal (Ovx) rat model. This model is widely used as an animal model to stimulate postmenopausal conditions related to estrogen deficiency, such as osteoporosis [12], cardiovascular diseases [13], and cognitive decline [14]. When promising results were obtained, we confirmed their effects through a double-blind randomized controlled trial in postmenopausal women [15,16].

Coconut (*Cocos nucifera* L.), cultivated primarily in tropical and subtropical regions [17], holds a significant place in traditional medicine, and its various components are reported to exert antibacterial, antidiabetic, anti-atherosclerotic, antioxidant, and hypolipidemic effects [17,18,19]. Despite its extensive use, there are cultural beliefs surrounding the impact of coconut juice on women’s health; it is known to disrupt their menstrual cycles [20], even serving as a temporary contraceptive in parts of Indonesia (Java). This perception extends to coconut milk, which some women avoid due to concerns about potential effects on fertility [21]. However, in Thai tradition, young coconut juice (YCJ) extracted from 6-month-old coconuts has been historically consumed to alleviate menopausal symptoms [22]. Based on the usage of YCJ as folk medicine, we speculated that it may have estrogenic properties. Although a prior study showed that 2.45 pg of 17β-estradiol (E2), along with other sex hormone-like substances, is included in 1 mL of YCJ [23], studies investigating the effects of YCJ on pathologies associated with estrogen deficiency using postmenopausal animal models are limited. Radenahmad et al. [21] demonstrated that wound healing was significantly accelerated in Ovx rats by YCJ at a dose of 100 mL/kg body weight daily for 5 weeks. In addition, we previously demonstrated that supplementation with YCJ for 6 weeks resulted in significantly higher femoral bone mass and indices of bone formation in Ovx rats [24] and that long-term (12-week) administration of YCJ alleviated body weight gain in a menopausal rat model [25].

The spectrum of menopausal symptoms affecting the central nervous system includes mood disturbances (anxiety and depression) and an effect on cognitive function (memory loss and cognitive difficulties). Memory deficits and depression-like behavior in Ovx rats were improved by the administration of E2 or phytoestrogen [26,27,28,29]. Therefore, our study aims to explore the influence of YCJ supplementation on memory impairment and depression-like behavior in Ovx rats.

## 2. Materials and Methods

### 2.1. Preparation of Young Coconut Juice

Dried YCJ powder was prepared from the fruit of coconuts that were approximately 6 months old (Hat Yai, Thailand), as previously described [24,25]. The powder was dissolved in distilled water to obtain 5×-concentrated YCJ. The final solution was stored at −20 °C until use.

The chemical composition of coconut juice has been reported in various studies [30,31], including key components such as sugars, electrocytes, vitamins, amino acids, and phytohormones. However, it is important to note that the composition can vary significantly depending on factors such as the coconut, the cultivation environment, and weather conditions.

### 2.2. Animal and Diet

Female Wistar rats (8 weeks old) were procured from a commercial vendor (Japan SLC Inc., Hamamatsu, Japan). The animals were acclimated in a controlled environment (temperature: 23 ± 1 °C, humidity: 55 ± 5%) with a 12 h light–dark cycle for 2 weeks. Throughout the study, the rats had ad libitum access to standard laboratory chow (MF; Oriental Yeast Co., Ltd., Tokyo, Japan) and distilled water. All the procedures were ethically reviewed and approved by the Animal Ethics Committee of the University of Shizuoka (Shizuoka, Japan), and the experiments were carried out following the established guidelines.

### 2.3. Protocol

Following the acclimation period, the rats were anesthetized using an intraperitoneal injection of a mixture of medetomidine hydrochloride (0.375 mg/kg body; FUJIFILM Wako Pure Chemical Co., Tokyo, Japan), midazolam (2 mg/kg body weight; FUJIFILM Wako Pure Chemical Co.), and butorphanol tartrate (2.5 mg/kg body weight; Meiji Seika Pharma Co., Ltd., Tokyo, Japan). They were then randomly allocated to the three groups: the Sham (sham-operated rats, n = 9), Ovx (rats undergoing bilateral ovariectomy via the dorsal approach, n = 10), and Ovx + YCJ groups (Ovx rats treated with 5×-concentrated YCJ by gavage at a dose of 15 mL/kg body weight—equivalent to 75 mL/kg body weight of 1× YCJ daily for 12 weeks, starting 2 days post-surgery, n = 9). The YCJ dosage was determined based on a prior study [21], ensuring that the serum E2 levels in the Ovx rats fed YCJ (20–100 mL/kg body weight) mirrored those in the sham-operated rats. The animals in the Sham and Ovx groups received distilled water by gavage every day for 12 weeks. One rat in the Ovx + YCJ group expired before the completion of the experiment for an unspecified reason. At the end of the 12-week period, the brain and uterus weights were measured during necropsy.

### 2.4. Spatial Learning and Memory Assessment Using Morris Water Maze Test

Nine to ten weeks post-surgery, the Morris water maze test was conducted to evaluate spatial learning and memory. This involved a circular vinyl pool (130 cm in diameter, 57 cm height) filled with clear tap water (22 ± 2 °C) to a height of 20 cm. Within the pool, a transparent circular platform (10 cm in diameter, 18 cm height) was placed 25 cm away from the pool wall, submerged below the water surface. Visual cues were placed around the room.

The rats were introduced into the pool and trained to locate the platform within a 40 s timeframe. In the event of failure, manual guidance to the platform was provided. Once the rats reached the platform, they remained there for 10 s before being placed in a holding cage. A block of four trials was conducted twice daily over three days. In each trial, the release points of the rats in the pool were changed randomly. During the first trial on day 1, the pool was divided into four quadrants, and rat movement across each quadrant was measured to evaluate motility, represented as times/latency time. Rat behavior was recorded using a video camera (Sony Co., Tokyo, Japan) and analyzed through ANY-maze commercial software version 5.3 (Stoelting Co., Wood Dale, IL, USA).

### 2.5. Depression-like Behavior Assessment Using Forced Swim Test

Eleven weeks after surgery, individual rats were forced to swim in an acrylic cylinder (18 cm in diameter, 30 cm height) filled with clear tap water (22 ± 2 °C) to a height of 22 cm. The animals were placed in the cylinder for 15 min, removed, and after a 24 h interval, subjected to another 5 min session. Their behaviors were recorded via a video camera (Sony Co.). Immobility time was measured from the point where rats were observed passively floating in water.

### 2.6. Statistical Analyses

GraphPad Prism software version 10.0.3 (GraphPad Software, Boston, MA, USA) facilitated all the data management and statistical analyses. The results are shown as the means ± standard error of the mean. Specific effects of Ovx and YCJ were assessed through one-way analysis of variance accompanied by Tukey’s multiple comparison test. Statistical significance was considered to be at a *p*-value below 0.05. Post hoc statistical power analyses were performed using G*Power software 3.1.9.7 for Windows (Heinrich-Heine-Universität Düsseldorf, Düsseldorf, Germany) with the level of significance set at α = 0.05.

## 3. Results

### 3.1. Impact of Young Coconut Juice on Rat Body, Uterus, and Brain Weights

**Figure 1:**
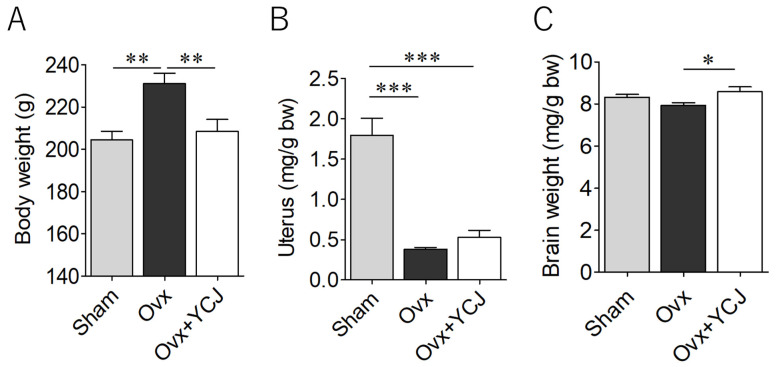
Body weight (**A**), ratio of uterus/body weight (**B**), and ratio of brain/body weight (**C**) among three groups: sham-operated (Sham), ovariectomized (Ovx), and Ovx rats supplemented with YCJ for 12 weeks (Ovx + YCJ). The values represent the mean ± SEM (n = 9–10 rats per group). * *p* < 0.05 and ** *p* < 0.01 compared with the Ovx group, and *** *p* < 0.001 compared with the Sham group (one-way ANOVA with Tukey’s multiple comparison test). Post hoc power was 0.97 (effect size 0.84) for (**A**), 1.00 (effect size 1.71) for (**B**), and 0.91 (effect size 0.74) for (**C**). YCJ: young coconut juice, SEM: standard error of the mean, ANOVA: analysis of variance.

As shown in our previous studies, Ovx rats exhibited an initial increase in body weight after surgery [24,25]. At the end of the experiment, the animals in the Ovx group demonstrated a significantly higher body weight (Figure 1A) and a notably lower uterus/body weight ratio (Figure 1B) compared to the Sham group, indicating the success of the surgical procedure. YCJ contributed significantly to the mitigation of the increase in body weight of the rats in the Ovx + YCJ group, although no specific impact on the uterus/body weight ratio was observed (Figure 1A,B). While not achieving statistical significance, the rats in the Ovx group displayed a lower brain/body weight ratio compared to the Sham group. Interestingly, the rats in the Ovx + YCJ group exhibited a noteworthy increase in the brain/body weight ratio, reaching levels akin to those of the sham-operated rats (Figure 1C).

### 3.2. Impact of Young Coconut Juice on Spatial Learning and Memory Evaluated via Morris Water Maze Test

**Figure 2:**
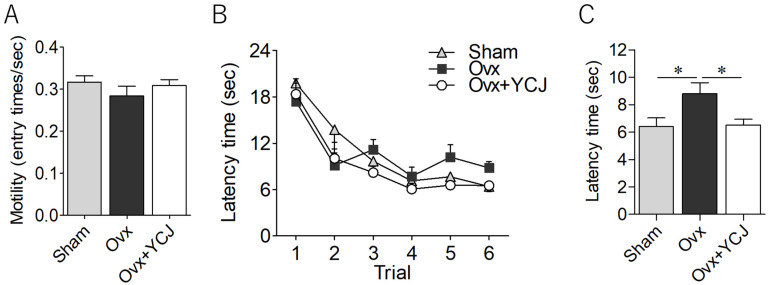
Assessment of motility based on entry times into each virtual quadrant zone divided by latency time (**A**), latency time until arriving at the platform over the course of trials (**B**), and latency time in the final set of trials (**C**) among three groups: sham-operated (Sham), ovariectomized (Ovx), and Ovx rats supplemented with YCJ for 12 weeks (Ovx + YCJ). The values represent the mean ± SEM (n = 9–10 rats per group). * *p* < 0.05 compared with the Ovx group (one-way ANOVA with Tukey’s multiple comparison test). Post hoc power was 0.21 (effect size 0.27) for (**A**) and 0.47 (effect size 0.43) for (**C**). YCJ: young coconut juice, SEM: standard error of the mean, ANOVA; analysis of variance.

To assess hippocampal-dependent spatial memory, the Morris water maze test was conducted. No significant difference in motility was found among the rats of the Sham, Ovx, and Ovx + YCJ groups during the first four trials (Figure 2A). The latency time to arrive at the hidden platform decreased over the successive trials (Figure 2B). Notably, during the final set of trials, the Ovx group exhibited significantly longer latency times compared to the Sham group, indicating impaired memory. However, the rats in the Ovx + YCJ group displayed improved memory performance, reaching levels comparable to those of the Sham group (Figure 2C).

### 3.3. Impact of Young Coconut Juice on Depression-like Behavior Evaluated via Forced Swim Test

**Figure 3:**
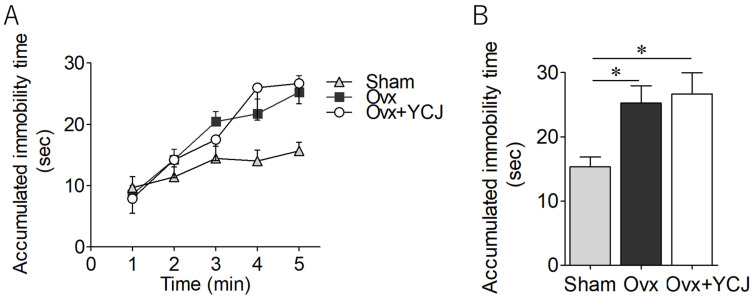
Accumulated immobility time over time (**A**) and for 5 min (**B**) among three groups: sham-operated (Sham), ovariectomized (Ovx), and Ovx rats supplemented with YCJ for 12 weeks (Ovx + YCJ). The values represent the SEM (n = 9–10 rats per group). * *p* < 0.05 compared with the Sham group (one-way ANOVA with Tukey’s multiple comparison test). Post hoc power was 0.56 (effect size 0.48). YCJ: young coconut juice, SEM: standard error of the mean, ANOVA; analysis of variance.

Depression-like behavior was assessed based on the immobility time in the forced swim test. While initial motility within the first 1 min showed no variation among the groups, both the Ovx and Ovx + YCJ groups exhibited a gradual increase in immobility time over the test duration (Figure 3A). At the cumulative 5 min mark, the rats in the Ovx group displayed significantly longer immobility times compared to the Sham group (Figure 3B). Conversely, the accumulated immobility time for the rats in the Ovx + YCJ group did not exhibit significant changes relative to the Sham group (Figure 3B).

## 4. Discussion

In Southeast Asia, women have traditionally turned to the consumption of YCJ to alleviate postmenopausal symptoms, hinting at the potential estrogenic properties in YCJ. However, research exploring the impact of YCJ on conditions linked to estrogen deficiency in postmenopausal animal models is limited. In the present investigation, we demonstrated that Ovx led to significant memory impairment in rats, as indicated by the results of the Morris water maze test. This impairment was alleviated by the administration of YCJ, resulting in the performance levels of the Ovx + YCJ rats being comparable to those of the sham-operated rats. However, YCJ treatment failed to ameliorate depression-like behavior in the Ovx rats, as evaluated using the forced swim test. These findings suggest that YCJ supplementation may help to enhance cognitive function but may not alleviate depressive mood in postmenopausal women, which contrasts with the effect of MHT. MHT has been shown to have limited effects on cognition. For example, the Women’s Health Initiative Memory Study reported that MHT did not improve cognitive function and that it may even increase the risk of dementia in certain cases [32,33]. However, MHT has been shown to be beneficial in improving depressive mood, particularly in early postmenopausal women [34,35]. Thus, while YCJ may have a more focused effect on cognition, MHT could be considered for its mood-enhancing effects.

The mechanisms by which YCJ supplementation may enhance cognitive function but not alleviate depressive mood following Ovx, and the reasons behind the difference in these results from the effects of MHT, are not fully understood. In a similar manner to those of the present study, the results of our previous study revealed that supplementing YCJ to Ovx rats for 12 weeks prevented body weight gain without affecting uterus weight [25]. In addition, we demonstrated that such treatment prevented periosteal apposition of femoral cortical bone, an effect mediated by estrogen via estrogen receptor (ER) β [36]. Considering the results of studies which indicated that the activation of ERβ improves memory deficits [26,37], we hypothesized that the effects of YCJ may be exerted, in large part, through ERβ, but not ERα, suggesting that the effects of YCJ may not be exerted, at least not solely, by 17β-E2 itself [25]. Pungmatharith [23] investigated sex hormone-like substances in YCJ and reported that YCJ contains several phytoestrogens, including β-sitosterol, α-spinasterol, stigmastatrienol, stigmasterol, and fucosterol. Among these, β-sitosterol accounts for 58% of the total composition in 1 mL of YCJ, making it the most abundant phytoestrogen. In contrast, the amount of 17β-E2, a primary estrogen hormone, was quite small, measured at only 2.45 ± 0.7 pg per 1 mL of YCJ. This significant difference in the quantities of phytoestrogen and 17β-E2 highlights the predominant role of phytoestrogens in YCJ’s composition, which could be responsible for its hormone-like effects. Phytoestrogens function differently from estrogen and act more like selective estrogen receptor modulators (SERMs), binding to ERα and ERβ and acting as agonists or antagonists depending on the compound itself, as well as the target tissue, via a higher affinity for ERβ [38,39]. Based on various investigations, Radenahmad et al. [22] speculated that YCJ may act as a type of SERMs for target tissues.

The reasons why the administration of YCJ did not improve depression-like behavior in the forced swim test remain speculative. In contrast, Rao and Rajam [40] reported that young coconut water significantly increased struggling time in the forced swim test; they employed a significantly lower dose of coconut water (4 mL/body weight) compared to the 15 mL/kg body weight used in our study. The investigators demonstrated decreased levels of 5-hydroxytryptamine (5HT) and its metabolite 5-hydroxyindoleacetic acid (5-HIAA), with an increased turnover (5HIAA/HT), and speculated that the antidepressant effect of coconut water may be exerted via homeostasis in monoamines synthesis. However, their study employed non-castrated adult albino mice and adult albino rats of either sex. Therefore, further studies are warranted to investigate of the effect of YCJ on depression-like behavior under various experimental conditions.

We are not yet certain about the exact constituents of YCJ that improved memory impairment in Ovx rats. However, we speculate that β-sitosterol may play a key role in improving memory deficits, as it constitutes more than half (58%) of the total composition of YCJ [23]. Some investigators have reported that herbal extracts, which are rich in β-sitosterol, ameliorate memory impairment in animal experiments [41,42]. Furthermore, β-sitosterol crosses the blood–brain barrier and inhibits the production of amyloid β (Aβ), the main component of neuritic plaques in Alzheimer’s disease [43,44]. Recently, Yadav et al. [45] reported that β-sitosterol treatment mitigated cognitive impairment and Aβ deposition in AlCl_3_-induced Alzheimer’s disease model mice. In addition, Ye et al. [46] reported that β-sitosterol treatment improved spatial learning and recognition memory ability in amyloid protein precursor/presenilin 1 double-transgenic mice. The investigators also found that β-sitosterol reduced the plaque load and suggested that such treatment may reduce the progression of Alzheimer’s disease.

Oxidative stress is an imbalance between free radicals and antioxidants in the body, and is implicated in the pathogenesis of various age-related diseases, including Alzheimer’s disease [47]. Menopause is associated with an increase in oxidative stress, making women more susceptible to such diseases after menopause. It is also notable that β-sitosterol shows powerful antioxidant properties and improves cognitive impairments, short-term memory problems, and locomotor limitations [48]. However, it has been reported that intestinal absorption of sitosterol is low, about one-tenth that of cholesterol in both rats and humans [49,50]. Previous studies indicated that YCJ also contains other phytosterols (stigmasterol, stigmastatrienol, α-spinasterol, and fucosterol) besides sitosterol, as well as flavonoids and phytohormones (abscisic acid, auxin, gibberellins, zeatin, etc.), which may contribute individually or synergistically to the beneficial effects of YCJ on brain dysfunction [51]. Further studies are needed to understand the mechanisms by which YCJ enhances cognitive function.

The present study also demonstrated that YCJ supplementation of Ovx rats prevented a decrease in the brain/body weight ratio. Studies that have investigated the effects of YCJ on histopathological changes in the brains of Ovx rats are limited. Radenahmad et al. administered YCJ to Ovx rats at a dose of 100 mL/kg body weight for 4 weeks and found a significant reduction in neuronal cell death [20] and Aβ deposition [52] in YCJ-treated Ovx rats compared to that in Ovx rats without treatment or to those treated with estradiol benzoate.

However, the findings should be interpreted with caution because of the following limitations of the study. First, probe tests were not conducted in the Morris water maze; so, the result was limited to the evaluation of escape latency. Second, we examined the effects of only a single dose of YCJ in Ovx rats. Phytoestrogens exert either an agonistic or antagonistic action on ERα and ERβ in a dose-dependent manner [53]. Although the administration of YCJ did not result in an improvement in depression-like behavior in our study, it has been reported that activation of ERβ improves depression-like behavior in Ovx rats [54,55]. Therefore, using different doses may have yielded different results. Third, the amount of YCJ administered to the animals (75 mL/kg body weight/day) for 12 weeks seems to be large; thus, the dosage cannot be simply applied to humans (equivalent to 3750 mL/day for a 50 kg women). In addition, it has been reported that the administration of YCJ to Ovx rats for 4 weeks (100 mL/kg body weight/day) caused unfavorable side effects, such as glycogen deposition in the liver, although such treatment delayed the pathologies associated with Alzheimer’s disease and prevented neuronal cell death [20,52,56]. Payanglee et al. [56] administered three different doses of YCJ daily (10, 20, and 40 mL/kg body weight) for 10 weeks to Ovx rats and found that the 10 mL/kg body weight was the best dose to preserve neuronal cell density in the hippocampus and prefrontal cortex, which constitute the regions responsible for learning and memory. In addition, the investigators reported that such a dosage (10 mL/kg body weight) did not affect the serum lipid profile or renal function at the end of the experiment, although it increased body weight and serum glucose in Ovx rats. Further studies are needed to establish experimental conditions that avoid adverse effects to further investigate the effects of YCJ in a postmenopausal rat model.

## 5. Conclusions

The administration of YCJ ameliorated the memory impairment of Ovx rats at a level that was comparable to that of the sham-operated rats, although it did not result in the improvement of depression-like behavior. These findings align with the fact that many postmenopausal women experience memory issues, which are often linked to hormonal changes during menopause [34,57]. The current study suggests that YCJ could be a promising therapeutic option for addressing memory deficits in postmenopausal women. This would provide an alternative for women who prefer natural products over conventional treatments. However, other therapeutic options, including MHT, should be considered to prevent the depressive moods observed after menopause.

Additionally, the broader effects of YCJ on other postmenopausal symptoms, such as vasomotor symptoms and cardiovascular health, remain poorly understood and warrant further investigation. There is currently no established daily intake of YCJ for postmenopausal women, and long-term safety studies are necessary. Further research should aim to determine the optimal dosage and further explore the potential of YCJ as a supplement to promote women’s health after menopause.

## Data Availability

The datasets generated and/or analyzed in the current study are available from the corresponding author upon reasonable request.
